# Exploring the potential of magnesium oxychloride, an amorphous magnesium phosphate, and newberyite as possible bone cement candidates

**DOI:** 10.1177/08853282231190908

**Published:** 2023-08-01

**Authors:** Friederike Kaiser, Lena Schröter, Philipp Wohlfahrt, Isabel Geroneit, Jérôme Murek, Philipp Stahlhut, Jan Weichhold, Anita Ignatius, Uwe Gbureck

**Affiliations:** 1 Department for Functional Materials in Medicine and Dentistry, 9190 University Hospital Würzburg, Würzburg, Germany; 2 Institute of Orthopedic Research and Biomechanics, 27197 Ulm University Medical Center, Ulm, Germany

**Keywords:** Bone cement, magnesium-based cements, resorbable, degradation, large animal model

## Abstract

Magnesium phosphate-based bone cements, particularly struvite (MgNH_4_PO_4_∙6H_2_O)-forming cements, have attracted increased scientific interest in recent years because they exhibit similar biocompatibility to hydroxyapatite while degrading much more rapidly in vivo. However, other magnesium-based minerals which might be promising are, to date, little studied. Therefore, in this study, we investigated three magnesium-based bone cements: a magnesium oxychloride cement (Mg_3_(OH)_5_Cl∙4H_2_O), an amorphous magnesium phosphate cement based on Mg_3_(PO_4_)_2_, MgO, and NaH_2_PO_4_, and a newberyite cement (MgHPO_4_·3H_2_O). Because it is not sufficiently clear from the literature to what extent these cements are suitable for clinical use, all of them were characterized and optimized regarding setting time, setting temperature, compressive strength and passive degradation in phosphate-buffered saline. Because the in vitro properties of the newberyite cement were most promising, it was orthotopically implanted into a partially weight-bearing tibial bone defect in sheep. The cement exhibited excellent biocompatibility and degraded more rapidly compared to a hydroxyapatite reference cement; after 4 months, 18% of the cement was degraded. We conclude that the newberyite cement was the most promising candidate of the investigated cements and has clear advantages over calcium phosphate cements, especially in terms of setting time and degradation behavior.

## Introduction

Synthetic bone cements based on calcium phosphates have been used for many years to treat critical bone defects and for bone augmentation. Bone cements are normally applied by mixing powder and liquid directly in the operating room (OR). This can be performed manually by the OR staff or semi-automated, for example, with double-chamber syringes. Depending on the type of cement and application, the resulting reactive cement is applied into the bone defect in a rather liquid form or as a kneadable mass. The cements typically set in less than 20 min after application and completely harden within a week, subsequently mechanically supporting the bone and avoiding fibrous tissue formation in the bone defect.

Scientific studies during the previous 15 years have shown that magnesium phosphate cements could provide a promising complement to synthetic calcium phosphate cements, because they display a similarly high biocompatibility while being resorbed more rapidly.^1,2^ Calcium phosphate cements, particularly those most frequently used in clinic that set to hydroxyapatite, are only slowly resorbed and can often still be found at the implant site after years.^3^ By contrast, magnesium phosphates have exhibited a rapid resorption, including distinct material replacement by new bone tissue within less than a year in small and large animal models.^4–9^ This suggests that magnesium phosphate cements are particularly promising for young patients with good bone regeneration.

To date, the most frequently investigated magnesium phosphate phase is struvite (MgNH_4_PO_4_·6H_2_O), which has displayed the aforementioned promising properties.^1,2^ However, apart from this, very few other magnesium phosphate phases have been investigated to date. Even though struvite has appeared to be very promising in vivo, released ammonium ions might have possible cytotoxic effects. Ammonium can be metabolized in the liver via the urea cycle, but higher ammonium ion concentrations may block docking sites of potassium transport proteins.^10,11^ This could pose a challenge for potential clinical approval. In a previous study, we investigated K-struvite (MgKPO_4_·6H_2_O) as a possible alternative, but this cement exhibited very rapid degradation, such that new bone formation was unable to sufficiently match the material degradation.^12^

Therefore, the aim of this study was to investigate three alternative cement types, which have been very sparsely explored in the literature to date, in terms of their suitability as bone cement materials: (1) magnesium oxychloride (Mg_3_(OH)_5_Cl∙4H_2_O), (2) an amorphous magnesium phosphate bone cement based on sodium dihydrogen phosphate, trimagnesium phosphate (TMgP, Mg_3_(PO_4_)_2_, farringtonite) and magnesium oxide (MgO); and (3) a newberyite cement (MgHPO_4_·3H_2_O). The advantage of these cements is that all possible released ions of the set cements occur in higher concentrations in the body: Mg^2+^, Cl^−^, Na^+^, PO_4_^3−^, O^2−^, HPO_4_^2−^, and H_2_PO_4_^−^, and, therefore, ion release during degradation of the cements is likely to be rather uncritical. In the following, the findings from the few studies that have already investigated these three cement types will be briefly summarized.

Magnesium oxychloride cements are already known from the construction industry but are very rarely used there due to their degradation in water, resulting in a decline of their mechanical stability. However, this might be ideal for a possible application within the body as a bone cement, provided that the degradation does not occur too rapidly. Tan et al. have already investigated a magnesium oxychloride cement as a possible biomaterial; here, phosphoric acid was added to improve the degradation stability of the cement.^13,14^ In contrast to the samples without phosphoric acid that already crumbled after 1 day in phosphate-buffered saline (PBS), the samples with 0.5 to 2 mol L^−1^ phosphoric acid displayed a weight loss of only approximately 12%–16% after 120 days. However, the additive resulted in a reduction of the compressive strength from 66 to 20–25 MPa.

The amorphous magnesium phosphate cement was developed by Mestres et al. and is formed by the reaction of magnesium oxide with sodium dihydrogen phosphate.^15^ Because no hydrate phase is formed during cement setting,^15^ this cement is referred to as “amorphous magnesium phosphate cement” hereinafter. Particular advantages of this cement are the high initial compressive strength of approximately 12–30 MPa after 1 h of setting in Ringer’s solution^15,16^ and antibacterial and bacteriostatic properties, which were demonstrated both in vitro by complete killing of *Streptococcus sanguinis* after a 9 h incubation of the bacterial solution with cement extract,^15^ and in vivo by decelerating osteomyelitis infection in rabbits caused by *Staphylococcus aureus*.^17^

Newberyite can be formed by the reaction of TMgP with phosphoric acid or magnesium dihydrogen phosphate dihydrate (MMPD) and water. Its high solubility (solubility product – log(K) at 25°C: 5.5–5.8)^18,19^ makes newberyite promising for a possible application as a bone cement, potentially leading to a rapid in vivo degradation. In our group, a calcium magnesium phosphate cement that sets to brushite and newberyite was developed more than a decade ago.^20^ The cement exhibited compressive strengths of 10–30 MPa,^20,21^ and biocompatibility was demonstrated with osteoblast-like MC3T3-E1 cells and by heterotopic implantation in rats.^20,21^ In vivo, the newberyite and brushite phases were completely dissolved after 15 months, but the less soluble whitlockite (Ca_9_(Mg)[PO_3_(OH)|(PO_4_)_6_]) was formed. Therefore, a calcium-free approach with a pure newberyite cement might be promising regarding degradation because the formation of low soluble calcium-containing precipitates is impeded. Pure newberyite has only been investigated, to date, in vitro in the form of three-dimensionally (3D) printed scaffolds,^22^ and in combination with silver as an antibacterial coating.^23^ To the best of our knowledge, no pure newberyite bone cement has been investigated in vitro or in vivo.

In this study, all three cement compositions were slightly adapted from the literature (when available), optimized regarding the setting temperature, compressive strength, and handling properties, and the phase composition was characterized. The most promising compositions were investigated regarding their passive degradation in PBS. Because the newberyite cement exhibited an optimal passive degradation compared to the other cement types, it was implanted in partly-loaded tibia bone defects in an ovine model with implantation times of 2 and 4 months. Biocompatibility, cement degradation and new bone formation were evaluated by histology, histomorphometry, and fluorochrome labelling.

## Experimental

### Cement fabrication

#### Magnesium oxychloride cement

For the cement setting experiments at the beginning of the study (Figure S1, Supporting Information), different magnesium oxides (MgO 22, MgO 27, MgO 291, MgO 2923) from the company Magnesia GmbH (Lüneburg, Germany) were mixed with MgCl_2_-solution (4 mol L^−1^, Merck KGaA, Darmstadt, Germany) in the respective powder-liquid-ratio (PLR). For the following experiments, only MgO 2923 was used for cement preparation since it exhibited the most rapid setting.

#### Amorphous magnesium phosphate cement

TMgP (Mg_3_(PO_4_)_2_) was synthesized by mixing MgHPO_4_·3H_2_O (Alfa Aeasar, Ward Hill, USA) and Mg(OH)_2_ (VWR International, Radnor, USA) in a molar ratio of 2:1 and sintering for 5 h at 1100°C. The resulting sinter cake was crushed and sieved to attain particle sizes of ≤355 µm.

The amorphous magnesium phosphate cement without TMgP was prepared by mixing MgO 2835 (Magnesia GmbH) and sodium dihydrogen phosphate (NaH_2_PO_4_, VWR International) in a molar ratio of 2.8:1. Ultrapure water was added as a cement liquid in the respective PLR. The amorphous magnesium phosphate cement with TMgP was prepared by adding 30 mol% TMgP (MgO + TMgP = 100 mol%) to the MgO (Table 1). The molar ratio between (MgO + TMgP) and the sodium dihydrogen phosphate was maintained at 2.8:1.

**Table 1.** Composition of the powder of the amorphous magnesium phosphate cement with 30 mol% TMgP.

	NaH_2_PO_4_	MgO 2835	TMgP
Molar mass (g mol^−1^)	120.0	40.3	262.9
Amount of substance (mmol)	10.0	19.6	8.4
Mass (g)	1.20	0.79	2.21

#### Newberyite cement

MMPD (Mg(H_2_PO_4_)_2_·2H_2_O) was synthesized, based on a patent by Wagh and Jeong,^24^ by slowly adding 3.2 g MgO 27 (Magnesia GmbH) to 9.825 mL H_3_PO_4_ (Merck KgaA) with simultaneous stirring. The product was dried overnight at 37°C, ground with pestle and mortar and additionally ground for 10 s in a coffee grinder. TMgP was synthesized as described for the amorphous magnesium phosphate cement.

TMgP and MMPD were mixed in a molar ratio of either 1.17:1 or 2.33:1. For the cement reaction, TMgP and MMPD were mixed with a spatula and phytic acid (Sigma-Aldrich, St.-Louis, US) with a concentration of 0.1 mol L^−1^ was added. For the final newberyite cement, which was also investigated in the in vivo study, a molar ratio of 2.33:1 (TMgP: MMPD) and a PLR of 2 g mL^−1^ was used. For the in *vivo study*, 4.82 g TMgP were mixed with a spatula with 2 g MMPD and 3.41 mL phytic acid (0.1 mol L^−1^). Before implantation, TMgP, MMPD and the phytic acid solution were gamma-sterilized with ≥ 25 kGy.

#### Calcium deficient hydroxyapatite cement

Calcium deficient hydroxyapatite (CDHA) reference cements were fabricated by mixing α-tricalcium phosphate (α-TCP) with a solution containing 0.083 M NaH_2_PO_4_ and 0.167 M Na_2_HPO_4_ with a PLR of 3 g mL^−1^ and incubating them for 3 days at 37°C and 100% humidity. α-TCP was fabricated by sintering monetite (CaHPO_4_, Honeywell Fluka, ThermoFisher Scientific, Waltham, USA) and calcite (CaCO_3_,Sigma-Aldrich, Merck KGaA, Darmstadt Germany) at stochiometric ratio for 5 h at 1400°C. After crushing the sinter cake, the resulting powder was milled for 2.5 h under dry conditions in a planetary ball mill PM 400 (Retsch, Haan, Germany).

### Cement characterization

The particle size of the different magnesium oxides (*n* = 3) was determined using a powder-isopropanol suspension by laser diffraction analysis (Horiba LA 300 Wet, Horiba, Kyōto, Japan).

For producing the hardened cement samples for in vitro testing, the respective cement paste was filled into silicone molds and hardened at 37°C and 100% humidity for 24 h.

For compressive strength evaluation, rectangular-shaped samples (*n* = 10) were fabricated in silicone molds with the dimensions 6 × 6 × 12 mm. Directly after hardening, samples were removed from the molds and finished with sandpaper. Compressive strength was determined using the static Universal Testing Machine Z010 (Zwick, Ulm, Germany) with a 10 kN load cell. Samples were measured upright with a crosshead speed of 1 mm min^−1^. Compressive strength in MPa was calculated by dividing the maximal force in *N* by the samples cross-sectional areas in mm^2^.

The passive degradation in vitro in PBS was determined by weight loss and inductively coupled plasma mass spectrometry (ICP-MS) measurements, similarly as described in a previous study.^12^ Cylindrical samples (*n* = 3) of the final cement compositions of all three cement types were fabricated with a height of 2 mm and a diameter of 5 mm in silicon molds with the respective size as described above. The resulting cylindrical cement samples were washed for 3 h in 300 µL PBS per sample and the washing solution was changed every hour to remove possible salt residues in the cement pores. Subsequently, samples were dried at 37°C overnight and the weight was determined. To avoid bacterial contamination during the degradation experiment, samples were disinfected for 1 day with 70% ethanol (500 µL per sample), which was changed three times. The experiment was conducted under sterile conditions. Samples were placed on a shaker (50 r/min) and incubated in 500 µL PBS per sample in Eppendorf tubes for 18 days at 37°C and the PBS was changed every 3 to 5 days. After 18 days, the samples were dried overnight at 37°C and the weight was determined again. Ion release in PBS was determined by ICP-MS (iCAP RQ, ThermoFisher Scientific) after a 1:50 dilution. The standard solutions contained 10, 1, 0.1, 0.01 and 0.001 mg L^−1^ Ca and 100, 10, 1, 0.1 and 0.01 mg L^−1^ Mg (Sigma-Aldrich, Merck KGaA). The respective ion concentration in PBS was subtracted for each sample.

X-ray diffraction (XRD) measurements (*n* = 3) were performed with a D8 Advance (Bruker AXS, Karlruhe, Germany) to analyze the phase composition. The in vitro samples were measured after setting of the respective cement for 24 h at 37°C and 100% humidity. The hardened samples of the magnesium oxychloride and newberyite cements were dried at 37°C for 12 h and crushed with pestle and mortar. The samples of the amorphous cement were directly crushed and not dried to avoid water loss, which would impede the correct determination of the amorphous content. XRD measurements were performed in polymethyl methacrylate sample holders, using copper K_α_-radiation, a power of the X-ray tube of 1600 W and a divergence slit of 2.5°. For all measurements, a step size of 0.02° and a sample rotation speed of 15 U min^−1^ were applied. The angle range and dwell time varied depending on the cement type: For the magnesium oxychloride cement it was 7–70° and 0.35 s/step, respectively. For the amorphous magnesium phosphate cement, an angle range of 10–80° and a dwell time of 0.35 s/step were used. For the quantification of the different newberyite cement compositions, measurements were performed over 10–80° with a dwell time of 0.35 s/step. All displayed XRD measurements of the newberyite cement, including the ones after implantation, were performed over 7–70°, with a dwell time of 1 s/step and samples were placed in silicon sample holders due to a smaller amount of available powder in the case of the explants. In vivo samples (*n* = 3) were prepared by removing fragments of the cement material from the implant area, drying them at 37°C and crushing them with pestle and mortar. Rietveld refinement for quantitative analysis was performed with the TOPAS 6.0 software (Bruker AXS). For the quantification, the structures ICDD #450948 (periclase), ICDD #70239 (brucite), ICSD #241169 (Mg_3_OH_5_Cl·4H_2_O), COD #9012534 (farringtonite), COD #9007632 (newberyite) and ICSD #30955 (MMPD) were used. The mass fraction of the crystalline parts (*c*_*j*_) of the amorphous magnesium cements were calculated with equation (1), according to Jansen et al.^25^

(1) $$c_{j}=s_{j}\;\frac{\rho_{j}V_{j}^{2}\mu_{j}^{*}}{G}$$

The respective unit-cell volumes (*V*_*j*_), densities (*ρ*_*j*_) and Rietveld scale factors (*s*_*j*_) of farringtonite or periclase were used directly from the structure files (.cif, .str) or determined with the TOPAS software. The total mass attenuation coefficient ( $\mu_{j}^{*}$ ) was calculated as the sum of the total mass attenuation coefficients of the raw materials, including the cement liquid water. For the attenuation coefficients of the raw materials, the absorption coefficient and the density were used. The instrumental constant (*G*) was determined according to equation (2) with the measurement of a corundum standard (ICDD #431484) with a defined crystalline mass fraction ( $c_{c}$ ). For the measurement, the exact same conditions as for the amorphous magnesium phosphate cement were applied. The corundum unit-cell volume (*V*_*c*_), density (*ρ*_*c*_) and Rietveld scale factor (*s*_*c*_) were used directly from the structure file or determined with the TOPAS software. The mass attenuation coefficient ( $\mu_{c}^{*}$ ) was calculated with the absorption coefficient and the density of corundum.

(2) $$G=s_{c}\;\frac{\rho_{c}V_{c}^{2}\mu_{c}^{*}}{c_{c}}$$

The amorphous content (*A*) in the samples was determined via equation (3), with *c*_*f*_ being the mass fraction of farringtonite and *c*_*p*_ being the mass fraction of periclase. All samples were measured in triplicate.

(3) $$A=1-\left(c_{f}+c_{p}\right)$$

Porosity of the newberyite cement samples (*n* = 3) was determined by mercury porosimetry after hardening of the samples for 24 h at 37°C and 100% humidity and subsequently drying for 12 h at 37°C. A two-step measurement with pore sizes between 3.6 nm and 1 mm was performed with a Pascal 140/440 (Porotec, Hofheim, Germany). The pore radius (*r*) is calculated as a function of the pressure ( p ) by the Porotec software via the Washburn equation (4). For this, a mercury contact angle (*Ѳ*) of 140° and a surface tension (*γ*) of 0.48 N m^−1^ are used for the calculation.

(4) $$r=\frac{2\gamma \cos Ѳ}{p}$$

The temperature during setting (*n* = 3) was measured with the thermometer from a magnetic stirrer (MR Hei-Tec, Heidolph Instruments GmbH & Co. KG, Schwabach, Germany). For each cement, 4.8–6 g cement powder were mixed with the respective cement solution. The temperature measurement was started 1 min after mixing and ended after 30 min. The temperature value was recorded every minute. The pH value (inoLab pH meter, Xylem Inc., Washington, US) was determined (*n* = 3) for 30 min after preparation of the respective cement and recorded every minute automatically by the device.

For backscattered electron (BSE) images and energy dispersive X-ray spectroscopy (EDX), one representative methyl methacrylate embedded section of each time point was coated with 4 nm platinum. BSE images were gained with a Zeiss Crossbeam 340 scanning electron microscope (Zeiss, Jena, Germany) at an acceleration voltage of 15 kV and a 20x magnification for the overview images, a 75x magnification for the detail images and a 35x magnification for the line scan images. For the EDX mappings and the line scan, the same SEM and acceleration voltage, and the EDX system X-Max 50 (Oxford Instruments, Wiesbaden, Germany) was used. The mappings were gained at a 75x magnification, the line scan at a 35x magnification, and the line scan was performed on a length of approximately 3270 µm.

### In vivo study

The animal experiment was approved by the local ethical committee (Regierungsprasidium Tubingen, Germany, no. 1451). All animal procedures were performed in accordance with the European Union Directive 2010/63/EU on the protection of animals used for scientific purposes. In total, 14 adult female merino sheep (age: 4–6 years, mean weight: 97 ± 9 kg) were used in the in vivo study. The animals were randomly assigned to two different surgical groups (each *n* = 7), depending on the implantation time (2 or 4 months). One animal from the 2 months group was excluded from the study because of an anesthetic incident in the OR. Each animal underwent surgery and received the newberyite cement randomly in one hind limb. The cements were implanted in the proximal tibia into a mechanically loaded trabecular bone defect, as described previously.^26,27^ A control group with empty defects was omitted in the present study, because the standardized bone defect was proven to be critical sized in a previous study^5^ and thereby the number of animals used in the experiment was kept to a minimum size, according to the 3Rs principle. The implantation of a clinically established reference cement was also omitted deliberately, because a CDHA cement as a clinical standard had already been investigated in a previous study in the same defect model using the same experimental procedure.^5^

The surgeries were conducted under general isoflurane anesthesia (Isofluran Baxter, Baxter GmbH, Unterschleißheim, Germany), which was induced by thiopental (5 mg kg^−1^ body weight; Thiopental Inresa®, Inresa GmbH, Freiburg, Germany) intravenously (i.v.). After a medial surgical approach to gain access to the proximal tibia, a standardized wedge-shaped bone defect (height: 6 mm, width: 14 mm, length: 24 mm) was created parallel to and 3 mm underneath the medial tibial plateau with a custom-made milling machine.^26^ Bone debris was removed under saline irrigation and the bone defect was dried with a swab. For cement application, the powder components (TMgP, MMPD) of the newberyite cement were mixed with the aqueous solution (phytic acid), the cement was applied into a syringe and subsequently injected into the bone defect. Once the cement was set, the defect was covered with a periosteal flap and finally the fascial, subcutaneous and skin tissue was routinely sutured in layers.

Intraoperatively the sheep received carprofen analgesia (4 mg kg^−1^ body weight subcutaneously (s.c.); Rimadyl®, Zoetis GmbH, Germany) and amoxicillin trihydrate (10 mg kg^−1^ body weight s. c.; Veyxyl® LA 20%, Veyx-Pharma GmbH, Schwarzenborn, Germany) for antibiotic prophylaxis, which was continued for 3 days postoperatively. For the dynamic assessment of new bone formation, two fluorochrome bone label injections were administered to each animal, with a time interval of 14 days: tetracycline hydrochloride (25 mg kg^−1^ body weight, i.v.; Ursocylcin®10% per injection, Medistar Arzneimittelvertrieb GmbH, Ascheberg, Germany) and calcein green (10 mg kg^−1^ body weight, i.v.; Sigma-Aldrich, Merck KGaA). The 2 months group received the fluorochromes at 4 and 6 weeks post-surgery, respectively, and the 4 months group at 11 and 13 weeks post-surgery, respectively.

The animals were sacrificed at two different evaluation time points of 2 and 4 months and the tibial bones with the implanted cement were collected for comprehensive post mortem analysis.

### Histological evaluation

The defect area of the proximal tibia with approximately 10 mm of adjacent bone was dissected, sawed into two halves and processed for histology. The medial part was used for non-decalcified histology, while the lateral part was processed for decalcified histology.

For non-decalcified histology, the medial part of each bone specimen was fixed in 4% buffered formaldehyde for 5 days, dehydrated in an ascending series of ethanol and embedded in methyl methacrylate (Merck KGaA). Thereafter, ground sections of approximately 90–110 μm were prepared, using the sawing and grinding technique developed by Donath and Breuner.^28^

The unstained sections were evaluated by fluorescence microscopy (Leica DMI6000B, Heerbrugg, Switzerland; filter cubes LED 405 and L5 ET for excitation wavelengths of 402 and 494 nm, respectively, both from Leica) to assess dynamic histomorphometry as described in the guidelines of the American Society for Bone and Mineral Research (ASBMR).^29^ A rectangular region of interest (ROI) of approximately 11 mm^2^ was defined adjacent to the central cement surface. In this ROI, representing an area where newly formed bone was replacing the degrading cement, the distance between the yellow tetracycline- and green calcein bone markers was determined to calculate the bone formation rate (BFR, *n* = 6 for 2 months, *n* = 7 for 4 months). Analysis was performed using histomorphometrical software (Osteomeasure™, Osteometrics Inc, Decatur, GA, USA).

Following fluorescence evaluation, the histological sections were stained with Giemsa according to standard protocols for histological evaluation under light microscopy (Leica DMI6000B). For histomorphometry (*n* = 6 for 2 months, *n* = 7 for 4 months), sections were scanned at 50-fold magnification. Using image-analysis software from Leica (Leica MMAF 1.4.0 MetaMorph Imaging System, Leica, Wetzlar, Germany), the relative amounts of cement (Cm.Ar/T.Ar), newly formed bone (B.Ar/T.Ar), and soft tissue (ST.Ar/T.Ar) per total tissue area were determined in a squared ROI (7.0 mm × 7.0 mm) centered in the defect area, as described elsewhere.^4,5,12^

The lateral part of each bone specimen was processed for decalcified histology. After decalcification in ethylenediaminetetraacetic acid (EDTA) for 3 months, the samples were embedded in paraffin and cut into 7 μm sections. Thereafter, sections were stained for tartrate-resistant acid phosphatase (TRAP) to visualize osteoclastic degradation of the cement implants. Cells with ≥ 3 nuclei, positive for TRAP-staining and located on the surface of cement or bone, were identified as osteoclasts. To determine the number of osteoclasts on the cement surface (*N*.Oc/Cm.Pm, *n* = 6 for 2 months, *n* = 7 for 4 months), cells were counted in six visual fields at the bone-implant interface under 200-fold magnification on each sample, using histomorphometrical software (Osteomeasure™, Osteometrics Inc., Decatur, GA, USA), as described previously.^12^

### Statistics

Data obtained in the in vivo study was tested for Gaussian distribution by Shapiro-Wilk test. All data was normally distributed and analyzed using unpaired *t* test. The level of significance was set at *p* < .05. Statistical analysis was performed using GraphPad Prism® (8.4.3, GraphPad Software Inc., La Jolla, CA, USA).

## Results and discussion

### Magnesium oxychloride cement

As in the publications of Tan et al.,^13,14^ magnesium chloride solution with a concentration of 4 mol/L was used for cement fabrication. However, phosphoric acid was not included, because it increased the initial setting times from 86 to 180–270 min.^13^ Because the initial setting time was still very long even without phosphoric acid, we first attempted to find a MgO that was as reactive as possible to shorten the setting time. For this purpose, different MgOs from the company Magnesia GmbH were compared (Figure S1, Supporting Information). For each one, the highest possible PLR was determined that still allowed paste formation to attain a setting time as short as possible. Subsequently, it was investigated how rapidly these cements set at 37°C and 100% humidity. Because MgO 2923 was the most reactive and exhibited a compressive strength of 7.8 MPa after 50 min of setting, this one was used in the further course of the study.

**Figure 1.**
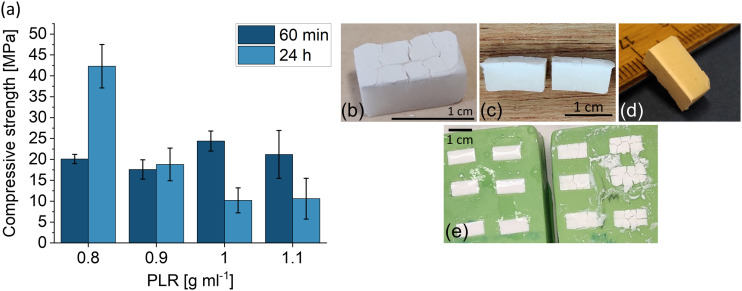
(a) Compressive strength of magnesium oxychloride cements with different PLRs (*n* = 10). (b)–(e) Images of magnesium oxychloride cements 24 h after setting at 37°C and 100% humidity in silicone molds. (b)–(d) PLR 1 g mL^−1^, (e) left mold: PLR 0.9 g mL^−1^, right mold: PLR 1.1 g mL^−1^.

After 1 h of setting, the compressive strengths of the cements prepared with the MgO 2923 were approximately 20 MPa, regardless of the PLR (Figure 1(a)). After 24 h of setting, surprisingly, the compressive strength decreased considerably from 42 to 10 MPa with increasing PLR. Normally, this relationship between PLR and mechanical performance is the opposite for cold-setting bone cements: With increasing PLR, the amount of liquid, which is normally in excess and acts as a pore forming agent, decreases and thus the porosity decreases and the compressive strength increases.^30^

**Figure 2.**
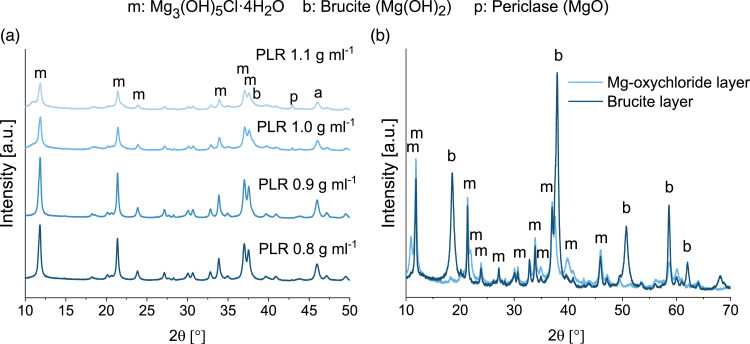
(a) XRD measurements of the set magnesium oxychloride cements with different PLRs. Although distinct brushite peaks were not visible, the peak shoulder at 38° broadened with increasing PLR, likely due to a slightly increasing brucite content. (b) XRD measurement of the separated white top layer (mainly brucite) and the rest of the cement (mainly magnesium oxychloride) of the magnesium oxychloride cement with a PLR of 1 g mL^−1^ after 24 h of setting.

In addition to the decreasing compressive strength, the formation of a white top layer was observed on the test specimens with a PLR of 1 and 1.1 g mL^−1^ after setting, which was not visible on the cements with a lower PLR (Figure 1(b)–(e)). This white surface layer also partially contained macroscopic cracks. XRD analysis showed that this surface layer consisted of brucite (Figure 2(b), Mg(OH)_2_). In the entire cement including the surface layer, the broadened peak shoulder at 38° in the XRD indicated a slightly increasing brucite content with increasing PLR (Figure 2(a)). According to equation (5), the PLR of 0.8 g mL^−1^ is stoichiometric, whereas a higher PLR results in MgO access, which hydrates to Mg(OH)_2_ and presumably negatively influenced the compressive strength. As stated by Walling et al., the hydration of MgO to Mg(OH)_2_ is accompanied by a considerable volume increase.^31^ Therefore, this white layer might have been formed due to excess MgO at the surface of the cement, and because it was the only area not covered by a silicone wall during setting, the Mg(OH)_2_ layer could be formed without restraint. Additional Mg(OH)_2_ formation within the cement might have resulted in tension or microcracks, subsequently resulting in a lower compressive strength.

(5) $$5MgO+MgCl_{2}+13H_{2}O\to 2Mg_{3}{\left(\mathrm{OH}\right)}_{5}Cl\cdot 4H_{2}O$$

Regardless of the PLR, the cements set mainly to Mg_3_(OH)_5_Cl∙4H_2_O, referred to as phase 5 in the literature (Figure 2, equation (5)).^31^ Because the composition with the PLR of 0.8 g mL^−1^ achieved the highest strength and did not exhibit the white, crack-containing brucite layer on top, it was chosen as the optimal composition of this cement and used for further investigations.

### Amorphous magnesium phosphate cement

**Figure 3.**
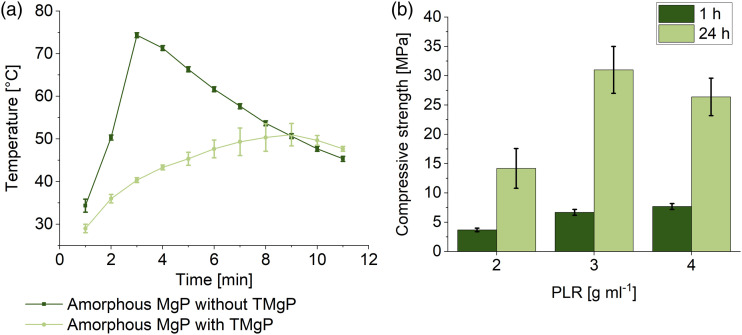
(a) Setting temperature of two amorphous magnesium phosphate cements with a PLR of 4 g mL^−1^ and with/without the addition of 30 mol% TMgP (percentage refers to the sum of TMgP and MgO = 100%) as an unreactive filler material (*n*=3). (b) Compressive strength of amorphous magnesium phosphate cements with different PLRs (*n* = 10). All compositions contained 30 mol% TMgP.

The composition studied by Mestres et al.^15^ was slightly modified here, and a molar MgO/salt ratio of 2.8:1 was used instead of 3.8:1. Similar to their study, we observed very high setting temperatures of up to 74°C (at PLR 4 g mL^−1^, Figure 3(a)). Mestres et al. solved this issue by adding 3 wt.% borax, thus the setting temperature was lowered from 61°C to 42°C. Because there is some evidence of reproductive toxicity of borax,^32^ it was not used here. Instead, the setting temperature was decreased to 51°C by adding 30 mol% TMgP. Because the TMgP was unmilled, it served as an unreactive filler material, which delayed the setting process. With lower TMgP contents the temperature was above 51°C and with higher TMgP contents an unwanted setting delay occurred. The compressive strength of these cements, fabricated with MgO, TMgP and NaH_2_PO_4_ (equation (6)), ranged from 14 to 31 MPa after 24 h of setting (Figure 3(b)), depending on the PLR. The decrease of the compressive strength from 31 MPa (PLR 3 g mL^−1^) to 14 MPa (PLR 2 g mL^−1^) with decreasing PLR was likely caused by an increase of porosity due to the higher liquid amount. Mestres et al.^15^ achieved overall higher compressive strengths of 30–50 MPa, either because of the higher PLR of 7.7 g mL^−1^, because of the absence of the unreactive filling material, or due to the higher MgO/salt ratio.

(6) $$MgO+{Mg}_{3}{\left({PO}_{4}\right)}_{2}+{NaH}_{2}PO_{4}+H_{2}O\to Amorphous\;product$$

**Figure 4.**
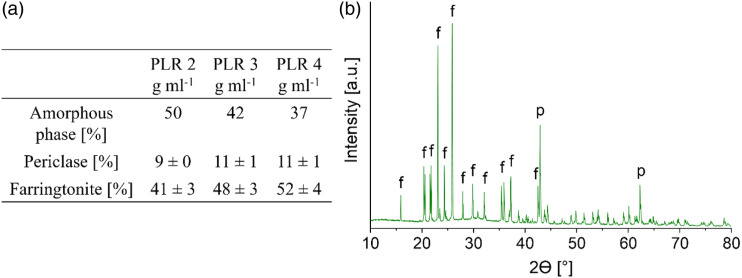
(a) Quantitative phase composition including the amorphous content, determined by XRD measurement, Rietfeld refinement and G-factor-method (*n* = 3). (b) XRD measurement of the amorphous magnesium phosphate cement with a PLR of 4 g mL^−1^. The crystalline part of the set cement contained only farringtonite (f) and periclase (p).

The crystalline parts in the set cement consisted only of the raw materials TMgP (farringtonite) and periclase (MgO) (Figure 4(b)). The cement reaction did not lead to the formation of a crystalline hydrate phase, instead, an amorphous, nanocrystalline or very poor crystalline phase was formed. The amorphous fraction was 37%–50% (Figure 4(a)) and decreased with increasing PLR. Presumably, the raw materials were increasingly in excess with increasing PLR, but there was also less water in the cement system, which is also X-ray amorphous. The PLR of 4 g mL^−1^ was used for all further investigations, since this cement exhibited the highest initial strength after 1 h and set fastest.

### Newberyite cement

**Figure 5.**
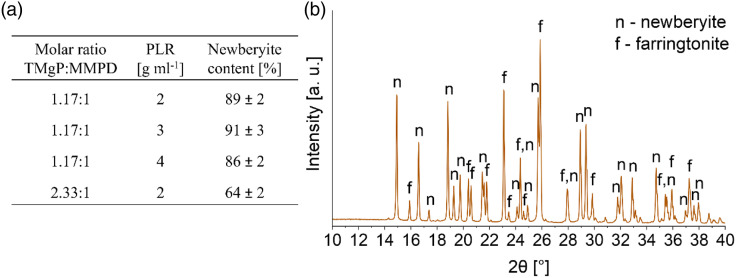
(a) Newberyite content depending on the molar ratio between TMgP and MMPD, and the PLR (*n* = 3). Values were determined by XRD measurements (*n* = 3) and quantitative analysis via Rietveld refinement. The phytic acid concentration in all compositions was 0.1 mol L^−1^. (b) XRD measurement of the newberyite cement with a molar TMgP-MMPD-ratio of 2.33:1 and a PLR of 2 g mL^−1^ after 24 h of setting at 37°C and 100% humidity.

The newberyite cement was prepared via the acid-base reaction of MMPD with TMgP (equation (7)). With a TMgP:MMPD ratio close to equimolar, a conversion degree to newberyite of 86%–91% was achieved (Figure 5(a)). The molar ratio of 1.17 was used because we originally thought to have synthesized Mg(H_2_PO_4_)_2_, and, therefore, used a mass ratio of 2.41, which refers to an exact equimolar ratio. Later, we found that the hydrate MMPD was synthesized instead of Mg(H_2_PO_4_)_2_, which corresponds at the same mass ratio of 2.41 to a molar ratio of 1.17.

(7) $$Mg{\left(H_{2}PO_{4}\right)}_{2}\cdot 2H_{2}O+Mg_{3}{\left(PO_{4}\right)}_{2}+10\;H_{2}O\to 4\;MgHPO_{4}\cdot 3H_{2}O$$

**Figure 6.**
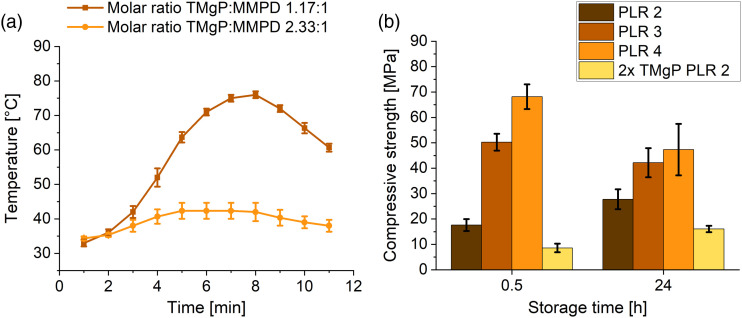
(a) Setting temperature of two newberyite cements with a PLR of 2 g mL^−1^ as a function of the molar ratio between TMgP and MMPD (*n* = 3). (b) Compressive strength of newberyite cements with different PLRs and different ratios between MMPD and TMgP (*n* = 10). Compressive strength after 24 h increased with increasing PLR for a molar TMgP-MMPD ratio of 1.17. The change of the molar TMgP-MMPD ratio to 2.33 (2x TMgP PLR 2) instead of 1.17 resulted in a strong decrease of the compressive strength.

Similar to the amorphous magnesium phosphate, the newberyite cement exhibited very high setting temperatures. If the cement was fabricated only with MMPD and TMgP with a molar ratio of 1.17, it set so rapidly that it did not even develop a pasty consistency. Therefore, phytic acid at a concentration of 0.1 mol L^−1^ was used as a setting retarder. It was previously demonstrated that 0.1 M phytic acid is well suited as a setting retarder for brushite cements and that the cytocompatibility with osteoblasts and osteoclasts is higher compared to citric acid.^33^ However, even with the phytic acid, the setting temperature was, with 76°C, very high (Figure 6(a)). The first attempt was to increase the phytic acid concentration, but this mainly resulted in a retarding effect, such that the high temperatures were reached just some minutes later. With 0.5 M phytic acid the setting temperature did not exceed 33°C over a time of 30 min, but the cement remained mechanically instable after 30 min. The next attempt was to use a TMgP excess to reduce the setting temperature. With a phytic acid concentration of 0.1 M and a molar TMgP:MMPD ratio of 2.33, which refers to the doubled amount of TMgP, the setting temperature could be reduced to 42°C (Figure 6(a)). For this composition, a PLR higher than 2 g mL^−1^ did not allow paste formation, therefore we chose the PLR of 2 g mL^−1^ to avoid increasing the porosity and thereby negatively impacting mechanical properties by using a lower PLR.

The TMgP excess (molar ratio 2.33) resulted in a lower conversion degree to newberyite of approximately 64 wt.% (Figure 5(a)), with the excess TMgP (farringtonite) being present in the final cement composition (Figure 5(b)). According to equation (7), with a TMgP:MMPD molar ratio of 2.33, the calculated newberyite content in the final cement should be 66.6 wt.%, which is only 2.6% more than we actually measured (Figure 5(a)). To achieve a rapid in vivo degradation of the material, we assumed that the newberyite content compared to the molar ratio of 1.17 is disadvantageous, because newberyite has a higher solubility than farringtonite.^18^ The compressive strength was also considerably reduced due to the great excess of TMgP from 28-47 MPa to 16 MPa (Figure 6(b)), and was thus also lower compared to Klammert et al., who achieved 30–40 MPa with the newberyite-brushite cements. However, because only this cement exhibited a sufficiently low setting temperature and the strength was acceptable, the composition with the TMgP:MMPD ratio of 2.33 and a PLR of 2 g mL^−1^ was used for all further investigations. The porosity of the final composition, determined by mercury porosimetry, was 32 ± 4% (Figure S2, Supporting Information).

### Comparison of the final compositions of the magnesium oxychloride, amorphous magnesium phosphate and newberyite cements

**Figure 7.**
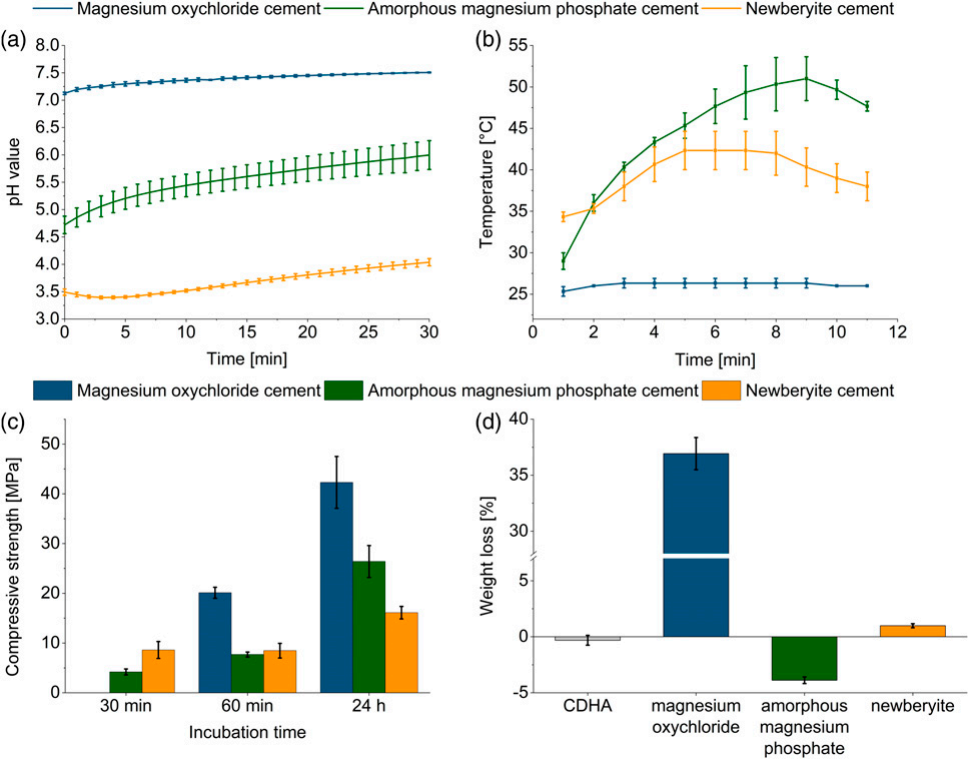
(a) pH value (*n* = 3), (b) temperature during setting (*n* = 3), (c) compressive strength (*n* = 10) and (d) weight loss after 18 days of storage in PBS (*n* = 3) of the magnesium oxychloride cement with a PLR of 0.8 g ml^–1^, the amorphous magnesium phosphate cement with a PLR of 4 g mL^−1^ and the newberyite cement with a molar TMgP-MMPD ratio of 2.33:1 and a PLR of 2 g mL^−1^. For the weight loss experiments, a CDHA reference cement was included.

For the optimum composition of the respective cements determined in this study, the pH value and temperature during setting, compressive strength and weight loss after 18 days of storage in PBS are presented in Figure 7. The magnesium oxychloride cement was the only cement with a physiological pH of 7.1–7.5 during setting (Figure 7(a)). The other two cements were within the acidic range, with pH values of 4.7–6.0 (amorphous magnesium phosphate cement) and 3.4–4.0 (newberyite cement). Although it would be ideal for bone cements to set at neutral pH, brushite cements, for example, were also found to be biocompatible even though they exhibit pH values between 2.5 and 5 during setting.^34^ Additionally, this low pH value is only shortly present during setting of the cement. The magnesium oxychloride cement featured a very low setting temperature of maximum 26°C (Figure 7(b)), matching the long setting time. Despite not even being rigid after 30 min, which made it impossible to measure the compressive strength, it exhibited a high initial compressive strength after 1 h of 20 MPa (Figure 7(c)). The amorphous magnesium phosphate cement reached a compressive strength of 4.2 MPa after 30 min at 37°C. The newberyite cement set most rapidly and exhibited already after 30 min a compressive strength of 9 MPa (Figure 7(c)). After a setting time of 24 h, the newberyite cement displayed, with 16 MPa, the lowest compressive strength, while the magnesium oxychloride cement exhibited, with 42 MPa, the highest strength. According to the aforementioned data, the amorphous magnesium phosphate and the newberyite cement appeared the most promising despite the lower strengths. Because the setting time is crucial for handling in the OR, the magnesium oxychloride cement was at a disadvantage here, owing to it still not being completely set after 30 min at 37°C. Despite the fact that we achieved an improvement of the setting time with the reactive MgO compared to the 86 min initial setting time reported by Tan et al.,^13^ the handling properties remained unacceptable for a possible clinical application as a bone cement.

**Figure 8.**
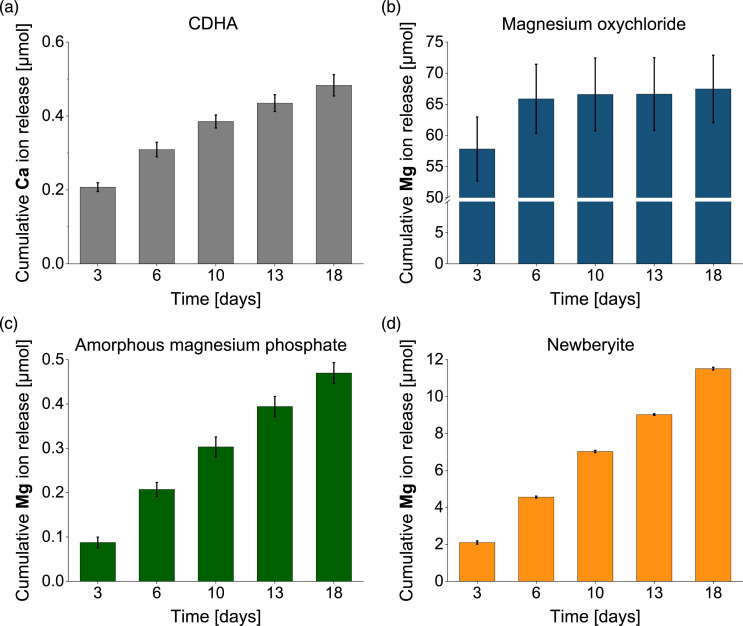
Cumulative calcium (Ca) or magnesium (Mg) ion release determined by ICP-MS after storage of cement samples over 18 days in PBS (*n* = 3). Investigated cements were: (a) CDHA reference cement (b) magnesium oxychloride cement with a PLR of 0.8 g mL^−1^, (c) amorphous magnesium phosphate cement with a PLR of 4 g mL^−1^ and (d) newberyite cement with a molar TMgP-MMPD ratio of 2:1 and a PLR of 2 g mL^−1^.

Regarding passive degradation in PBS (Figure 7(d) and 8) of the set cements, by contrast, the newberyite cement was the one that exhibited the most desirable degradation behavior (Figure 8(d)). Although the weight loss after 18 days was low at 1% (Figure 7(d)), the cumulative magnesium ion (Mg^2+^) release (12 µmol) was more than 10-fold higher than the cumulative calcium ion (Ca^2+^) release from the CDHA reference cement (0.5 µmol). Klammert et al. even observed a cumulative release of approximately 70 μmol Mg^2+^ from a newberyite-brushite cement after 21 days in cell culture medium, but they also had larger samples (Ø=15 mm, height: 1.5 mm).^20^ Gefel et al., who had a similar sample size as in our study, reported a cumulative Mg^2+^ release of approximately 5 µmol of 3D-printed macroporous newberyite scaffolds after 21 days in cell culture medium.^22^ When comparing the values of these two studies^20,22^ with ours in mmol L^−1^, they are in a similar range: 24 mmol L^−1^ (our study), 35 mmol L^−1^ and 25 mmol L^−1^.

The Mg^2+^ release (0.5 µmol = 1 mmol L^−1^) of the amorphous magnesium phosphate cement (Figure 8(c)) was comparable to the Ca^2+^ release of the CDHA (Figure 8(a)), and the material also displayed a mass increase of 4%. This was contradictory to Mestres et al., who reported a weight loss of approximately 2% and a Mg^2+^ release of approximately 70 µmol after 20 days of storage in PBS.^16^ However, their samples (Ø = 15 mm, h = 2 mm) had a threefold greater diameter compared to our samples, and they used 50 mL PBS, resulting in a Mg^2+^ release of 1.4 mmol L^–1^, which was comparable to ours.

The magnesium oxychloride cement degraded much too rapidly, with a mass loss of 37% and a Mg^2+^ release of 67 µmol after 18 days (Figure 7(d) and 8(b)). There was a burst Mg^2+^ ion release within the first 3 days of 58 µmol, likely due to the rapid dissolution of the phase 5, which was reported to be unstable in solutions with a Mg molality of <1.47 mol kg^−1^.^31^ The considerable mass loss was also partly due to the detachment of smaller cement particles, that is, not solely due to chemical dissolution, but also because of mechanical disintegration of the cement. By contrast, Tan et al. achieved with the phosphoric acid stabilization a weight loss of only 11%–17% in PBS after 120 days, which would be comparable to 1.7%–2.6% in 18 days.^13,14^ Because only the newberyite cement exhibited a promising degradation behavior, it was implanted in partly mechanically loaded tibial defects in an ovine model with implantation time points of 2 and 4 months.

### In vivo animal study

#### Clinical and macroscopical observations

No complications were observed in the post-surgical period. The animals returned to their physiological walking gait within approximately 3 days and displayed no movement anomalies in the further course of the animal experiment. Wound healing proceeded uneventfully without macroscopical signs of inflammation or infection. When the specimens were harvested after 2 or 4 months, no obvious inflammatory reaction was observed. The cements displayed a tight integration with the surrounding bone and the medial implant surface was covered with a thin layer of newly formed bone.

#### Histological analysis

**Figure 9.**
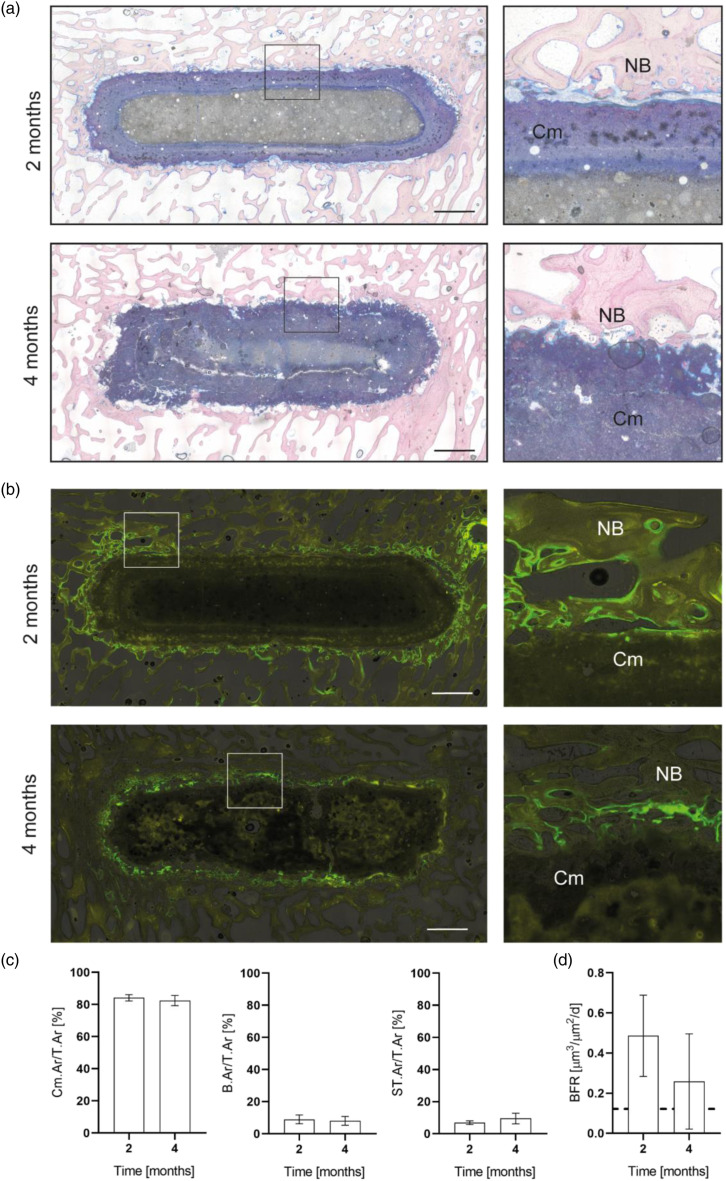
(a) Representative histological images (giemsa staining) and (b) fluorescence images of the newberyite cement at 2 and 4 months after implantation into ovine tibial bone defects. Overviews and magnifications of the bone-implant interface. Scale bar 2.5 mm. Cm – newberyite cement, NB – newly formed bone. (c) Relative cement, bone and soft-tissue area at 2 and 4 months after implantation. Cement area per total tissue area (Cm.Ar/T.Ar), bone area per total tissue area (B.Ar/T.Ar), and soft tissue per total tissue area (ST.Ar/T.Ar), *n* = 6 for 2 months, *n* = 7 for 4 months. (d) Bone formation rate (BFR) of fluorescence-labelled bone adjacent to the cement. Dashed line = BFR of surrounding intact trabecular bone, *n* = 6 for 2 months, *n* = 7 for 4 months.

Evaluation of Giemsa-stained histological sections revealed no inflammatory or infectious processes or signs for any foreign body reaction 2 or 4 months after implantation of the newberyite cement into tibial bone defects (Figure 9(a)). This supported the results of other studies that have evaluated magnesium phosphates and reported a good biocompatibility both in vitro and in vivo*.*^4–6,12,35–37^

Interestingly, after 2 months of implantation, the newberyite cement appeared double-layered, with an outer, blue-stained layer and an inner greyish layer. Over the time course to 4 months, the inner gray layer was diminished, while the blue layer appeared to increase from the outside to the inside in a centripetal manner, indicating a change in the composition of the cement due to a degradation process. Moreover, the outer edges of the cement appeared more irregular and dispersed at 4 compared to 2 months after implantation and released cement particles were observed close to the cement surface between adjacent bone trabeculae. Quantitative histomorphometrical analysis showed no significant changes in the relative cement area per total tissue area (Cm.Ar./T.Ar, Figure 9(c)) between 2 (84%) and 4 months (82%). In a previous study of our group with the same defect model, and under similar experimental conditions, the control hydroxyapatite cement showed basically no degradation even 10 months after implantation.^5^ However, the degradation of the newberyite cement here was slower compared to struvite forming cements, for which a residual struvite cement content of 64 or 75% was observed after 4 months in predecessor studies with the same defect.^5,12^ The slower in vivo degradation of the newberyite cement compared to struvite was surprising, because the in vitro properties were similar, particularly the weight loss after 18 days in PBS (1%, same experimental conditions, Figure 7(d)) and the cumulative magnesium ion release over 18 days in PBS (10–12 µmol, same experimental conditions, Figure 8(d)).^12^ Additionally, the solubility of newberyite in water at 25°C is, with 1690–2540 mg L^−1^, even higher compared to struvite (8–119 mg L^−1^).^18,19,38,39^ It might be that the remaining farringtonite raw powder particles exhibited a very slow degradation (see *Phase composition and elemental analysis of explants*), resulting in an overall reduced degradation of the cement.

**Figure 10.**
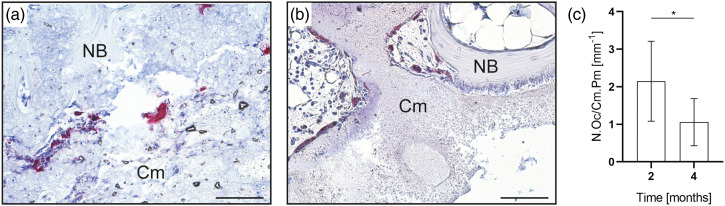
Histological analysis of paraffin sections stained for TRAP. Representative images of the bone-cement interface at (a) 2 months and (b) 4 months after implantation. Cm – newberyite cement. NB – newly formed bone. 200x magnification. (c) Number of osteoclasts located on the newberyite cement surface, indicating active resorption. N.Oc/Cm Pm – osteoclast number per cement perimeter. *p < .05, *n* = 6 for 2 months, *n* = 7 for 4 months.

Osteoclastic cells were observed in paraffin sections stained for TRAP to be located on the cement surface, suggesting additional active resorption of the newberyite cement (Figure 10). However, because cellular degradation is mainly limited to the bone-implant interface, it occurs much slower compared to passive dissolution. Moreover, the number of osteoclasts on the cement surface decreased significantly over time (Figure 10), indicating a diminishing cellular resorption over time.

The newberyite cement exhibited a good osseointegration after both 2 and 4 months, with regular direct bone-to-cement contact (Figure 9(a)). Osteoid was observed on the surface of the implants, produced by palisades of active osteoblasts. In addition, pre-osteoblastic cells as well as phagocytes and newly formed capillary vessels were found close to the cement surface. No significant changes were found in the relative amount of bone (B.Ar/T.Ar) or soft tissue (ST.Ar/T.Ar) between 2 and 4 months (Figure 9(c)), which can be attributed to the rather slow degradation of the newberyite cement.

Qualitative fluorescence microscopy revealed an overall higher fluorescence intensity of the bone directly adjacent to the implant, indicating increased bone formation compared to the surrounding intact trabecular bone (Figure 9(b)). In accordance with these observations an increased BFR was quantitatively determined for the bone next to the cement surface (Figure 9(d)), with a trend to decrease between 2 and 4 months post-surgery, but without statistical significance. In combination with a significantly decreasing number of osteoclasts on the cement surface, this suggests that the overall degradation and new bone formation process might have declined increasingly over time.

#### Phase composition and elemental analysis of explants

**Figure 11.**
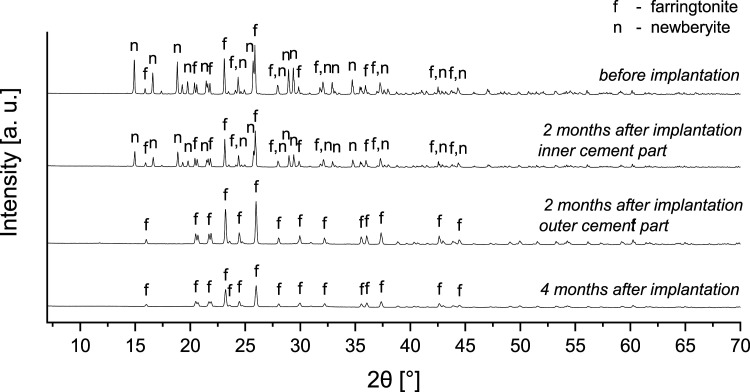
Phase composition determined by XRD before and 2 and 4 months after implantation. After 2 months, the remaining cement consisted of an inner and an outer layer, therefore, both layers were prepared individually for measurement. After 4 months, this layered structure was absent and only farringtonite remained.

After 2 months, in four of six samples a form of bilayer was observed macroscopically in the remaining cement in the defect area, with a brighter part in the middle and a slightly darker part at the rim (Figure S3, Supporting Information). The double-layered appearance of the cement implant at the earlier investigation time point was also observed histologically, as described earlier. For three independent samples, the two areas were prepared separately and examined by XRD. It was found that the inner part was a mixture of farringtonite and newberyite, comparable to the original material, while in the outer part the only crystalline component was farringtonite (Figure 11). This was reproducible for all three samples measured (Figure S4, Supporting Information). After 4 months, these two areas were no longer macroscopically recognizable (Figure S3, Supporting Information), therefore, the entire cement residue was prepared for analysis. Again, three samples were prepared and here, only farringtonite was recognizable as a crystalline component in the XRD, just as it was after 2 months in the outer layer (Figures 11 and S4, Supporting Information). This indicates that the newberyite phase was slowly dissolved out of the cement from the outside to the inside, and newberyite, as expected, exhibited a higher solubility in vivo compared to farringtonite, likely due to the higher passive solubility of newberyite. The solubility products of newberyite (-log K: 5.5–5.8) and farringtonite (23.4) correspond to solubilities of 1690–2540 mg L^−1^ (newberyite) and 2.2 mg L^−1^ (farringtonite) reported at 25°C.^18,19^

It was surprising that the overall degradation of the cement was rather slow, as observed histomorphometrically, although the newberyite phase was completely dissolved after 4 months. It seems that the remaining farringtonite phase degraded to a very limited extend. This might be attributed to the fact that the farringtonite raw powder was not additionally milled after sintering to avoid high temperatures during setting, resulting in larger farringtonite particles which degraded only slowly. By contrast, the farringtonite raw powder used for the fabrication of the struvite cements,^5,12^ for which we observed a more rapid degradation in vivo, was additionally milled after sintering, resulting in smaller particles that likely degraded more easily.

To the best of our knowledge, only one study, by Klammert et al., has investigated newberyite cements in vivo.^21^ Although in this study the XRD measurements of the cement remnants were only performed 15 months after implantation, considerably later than in the present study, they also revealed complete dissolution of the newberyite phase.^21^ Because the cement consisted initially of a mixture of newberyite and brushite, the main crystalline phase remaining after 15 months was whitlockite (Ca_18_Mg_2_(HPO_4_)_2_(PO_4_)_12_), which exhibits a low solubility. In addition, the newberyite-brushite cement displayed a very interesting dissolution behavior: on micro-computed tomography (µ-CT) images 15 months after implantation the core of the cement body was almost empty, whereas the outer part displayed only minor degradation.^21^ The authors suggested that this might have been caused by an inner dissolution of the material providing calcium, magnesium and phosphate ions, which subsequently precipitated at the outside of the cement as whitlockite due to the contact with the extracellular medium, which lead to oversaturation. This degradation behavior appears contradictory to the observations in the present study, but was likely caused by the presence of calcium ions in the cement and the heterotopic implantation in contrast to the orthotopic implantation here. As hypothesized, the absence of calcium ions in the cement composition here impeded the precipitation of low soluble calcium phosphates.

The double-layered appearance of the cement was also observed in the BSE images (Figures S5 and S6, Supporting Information), even 4 months after implantation a small inner layer was still recognizable. In the bone close to the cement surface only a few small magnesium EDX signals with a size of 10–20 µm were detected 2 and 4 months after implantation, possibly belonging to small remaining fragments of the already resorbed cement (Figure S5, Supporting Information). An EDX line scan across the inner and outer layers (Figure S6, Supporting Information) in the cement revealed that the Mg and P content was approximately 4–7% greater in the inner layer than in the outer layer (Table S1), presumably due to dissolution of the newberyite phase in the outer layer, which likely increased porosity. In addition, the Mg/P mass ratio was at both timepoints greater in the outer layer than in the inner layer, probably also due to dissolution from the newberyite phase (Table S1).

## Conclusions

In the present study, three novel cement systems were investigated with the aim of developing rapidly degrading, ammonium ion-free bone cements: magnesium oxychloride, an amorphous magnesium phosphate cement, and newberyite. Although the magnesium oxychloride cement with 42 MPa achieved the greatest strength of all the studied cements, and the setting time was shortened compared to the study of Tan et al.,^13^ the still long setting time (> 30 min) and extremely rapid passive degradation are disadvantages for a possible application as a bone cement. Here, further research on additives that both reduce the degradation rate and shorten the setting time needs to be conducted. Amorphous magnesium phosphate cements appear promising, particularly because of the antibacterial properties reported by Mestres et al.^15,17^ However, we observed no degradation and little ion release over 18 days in PBS, suggesting a low passive solubility of this cement.

In terms of passive degradation in PBS, the newberyite cement was the most promising material. Here, for the first time, a pure newberyite cement was investigated, and the absence of calcium ions successfully prevented the precipitation of less soluble calcium phosphate phases, such as whitlockite. When orthotopically implanted in sheep, the cement displayed excellent biocompatibility and degraded more rapidly compared to a hydroxyapatite reference cement, with 82% of the cement still present after 4 months. In conclusion, the newberyite cement was the most promising candidate of the investigated cements and has clear advantages over calcium phosphate cements, especially in terms of setting time and degradation behavior.

## Supplemental Material

Supplemental Material - Exploring the potential of magnesium oxychloride, an amorphous magnesium phosphate, and newberyite as possible bone cement candidates Click here for additional data file. Supplemental Material for Exploring the potential of magnesium oxychloride, an amorphous magnesium phosphate, and newberyite as possible bone cement candidates by Friederike Kaiser, Lena Schröter, Philipp Wohlfahrt, Isabel Geroneit, Jérôme Murek, Philipp Stahlhut, Jan Weichhold, Anita Ignatius, and Uwe Gbureck in Journal of Biomaterials Applications.
