# Supplementary material for: Exploring the potential of magnesium oxychloride, an amorphous magnesium phosphate, and newberyite as possible bone cement candidates
Source: J Biomater Appl. 2023 Aug 1;38(3):438–54. doi: 10.1177/08853282231190908 (PMC10494481; doi:10.1177/08853282231190908)
Supplement: Supplemental Material - Exploring the potential of magnesium oxychloride, an amorphous magnesium phosphate, and newberyite as possible bone cement candidates [file sj-pdf-1-jba-10.1177_08853282231190908.pdf]

Supporting Information

Exploring the potential of magnesium oxychloride, an amorphous magnesium phosphate, and newberyite as possible bone cement candidates

Friederike Kaiser[1*], Lena Schröter[2*], Philipp Wohlfahrt[1], Isabel Geroneit[1], Jérôme Murek[1], Philipp Stahlhut[1], Jan Weichhold[1], Anita Ignatius[2$], Uwe Gbureck[1$]

[1] Department for Functional Materials in Medicine and Dentistry, University Hospital Würzburg, Pleicherwall 2, D-97070 Würzburg, Germany

[2] Institute for Orthopedic Research and Biomechanics, Ulm University Medical Center, Helmholtzstraße 14, D-89081 Ulm, Germany

* The first two authors contributed equally to the study.

[$] Both senior authors contributed equally to the study.

(a)

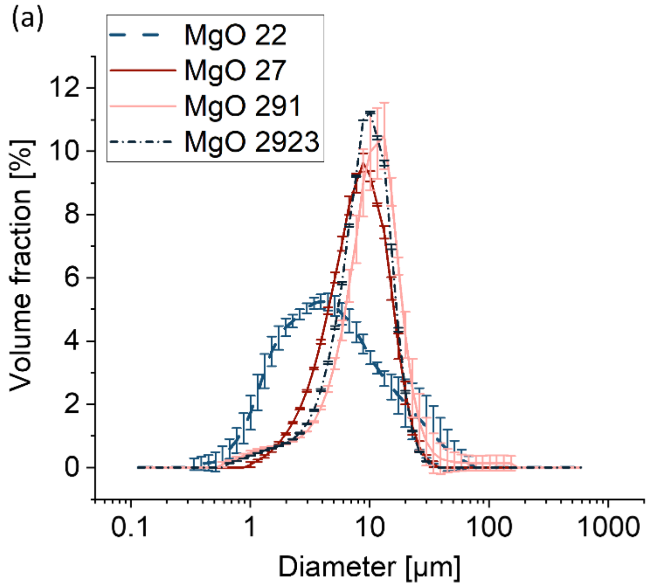

(b)

MgO	Particle size (d50 value) in µm	PLR in g ml^{-1}	Solidification time
22	4.2	1.6	≥2 h
27	7.6	1.3	≥2 h
291	9.5	1.4	≥2 h
2923	8.5	1.3	35 min

Figure S1. (a) Particle size distribution (n=3) of different commercially available magnesium oxides (company Magnesia GmbH, Lüneburg, Germany). (b) Results of the tentative setting experiments with the different MgOs and setting at 37°C and 100% humidity. The MgO 2923 was the most reactive one.

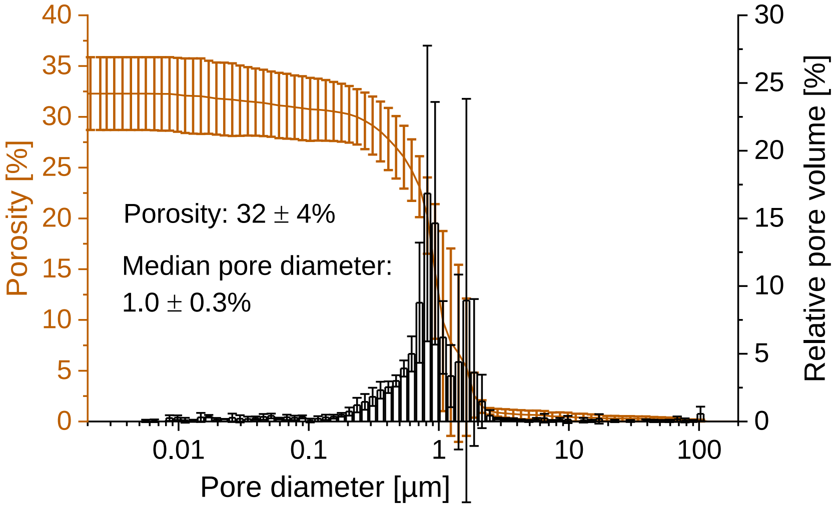

Figure S2. Cumulative porosity (left y-axis) and histogram of relative pore volume (right y-axis) of the newberyite cement with a molar TMgP-MMPD -ratio of 2:1 and a PLR of 2 g ml^{-1} after 24 h of setting at 37°C and 100% humidity (n=3).

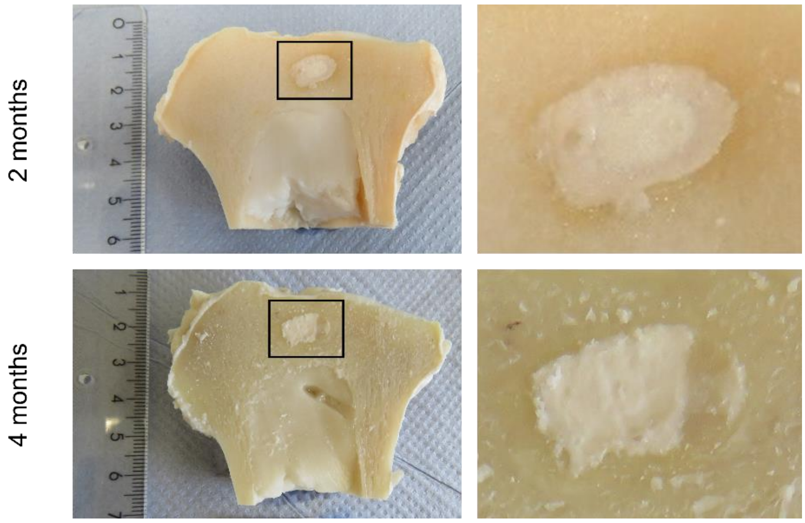

Figure S3. Images of the tibia samples after 2 and 4 months before preparation of the samples for XRD measurements.

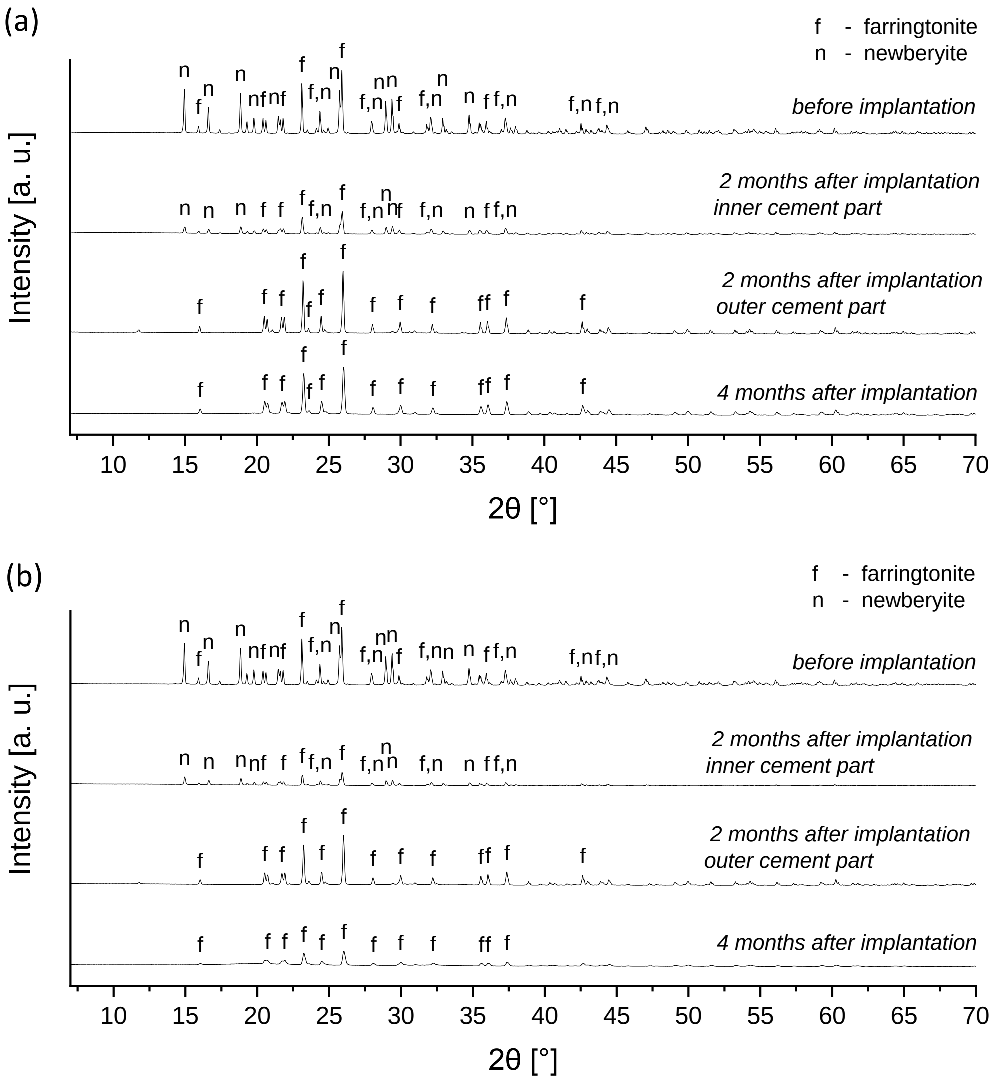

Figure S4. Phase composition determined by XRD before and 2 and 4 months after implantation. (a) and (b) present measurements of different samples.

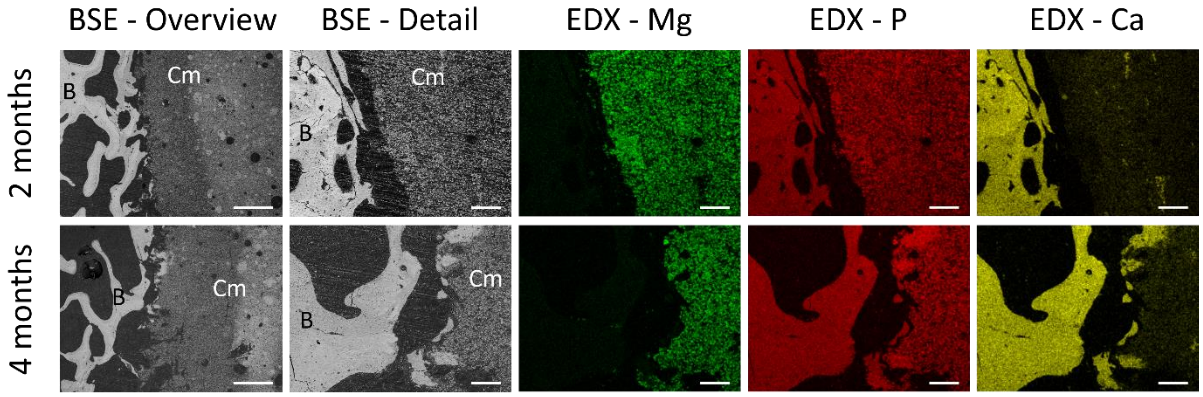

Figure S5. BSE images and EDX mappings of magnesium (Mg), phosphor (P) and calcium (Ca) of the transition zone between implanted cement (Cm) and bone tissue (B) 2 and 4 months after implantation. The EDX mappings correspond to the respective BSE-Detail image. Scale bar of the overview image: 1000 µm, scale bar of the detail image and the EDX mappings: 200 µm.

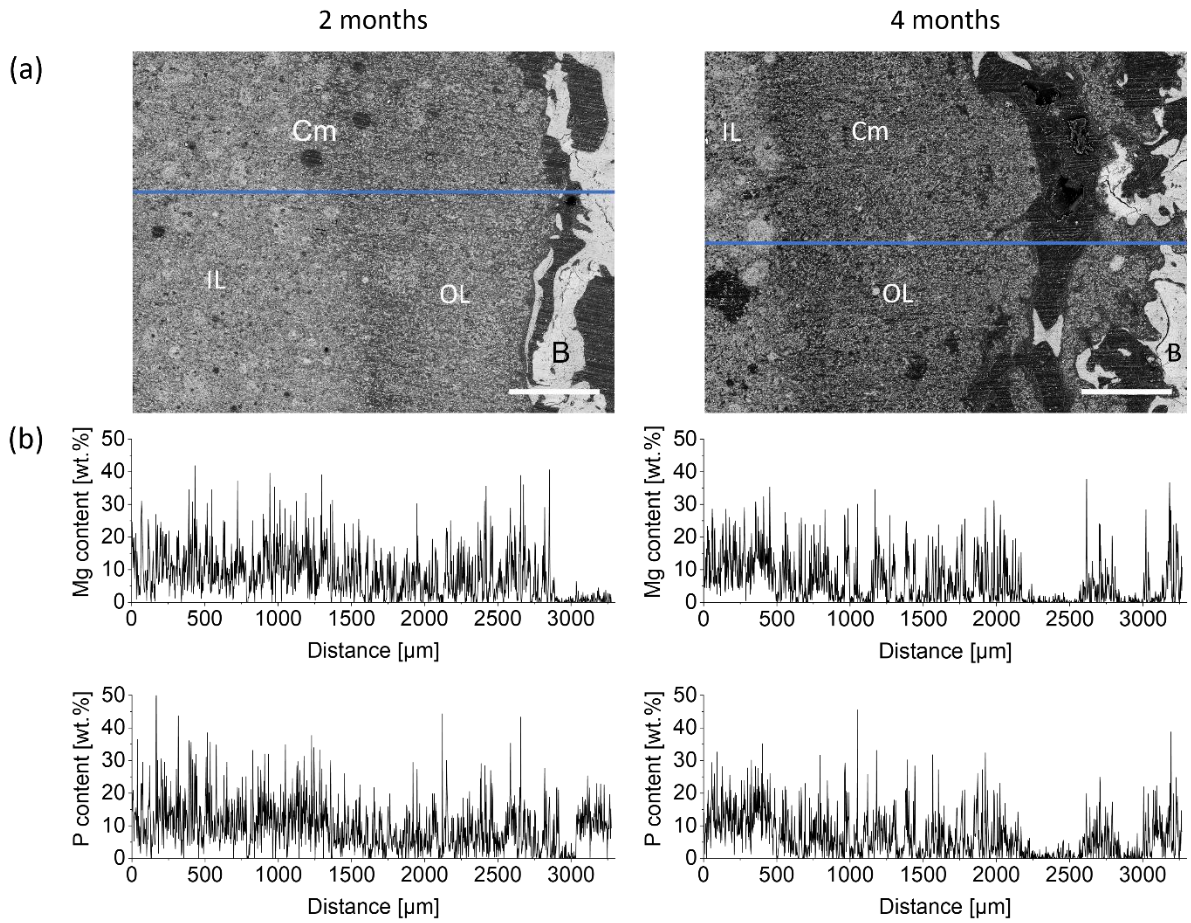

Figure S6. (a) BSE images of the implantation zone 2 and 4 months after implantation and line scan position (blue line). Two layers were visible in the remaining cement, labelled as inner layer (IL) and outer layer (OL). Scale bar 600 µm. (b) EDX line scan plots of the magnesium (Mg) and phosphor (P) content along the blue line in (a). The lines started in the center of the implant covering the two cement (Cm) layers and ended in the bone (B).

Table S1. Mean magnesium (Mg) and phosphor (P) content, and mean Mg/P ratio along the inner layer (IL) and outer layer (OL) cement parts in the line scans from Figure S5. The theoretical Mg/P mass ratio of farringtonite is 1.16, and the theoretical Mg/P ratio from the implanted material (64 wt.% $MgHPO_4 \cdot 3H_2O$, 36 wt.% $Mg_3(PO_4)_2$) is 0.93.

Position	Distance range [µm]	Timepoint	N	Mg content [wt.%]	P content [wt.%]	Mg/P mass ratio
IL	0–1304	2 months	598	11.3±7.1	12.6±7.7	0.89±0.02
OL	1306–2896	2 months	729	7.3±6.8	7.5±6.2	0.98±0.10
IL	0–478	4 months	220	12.5±6.6	13.1±6.3	0.95±0.05
OL	479–2204	4 months	792	6.0±6.5	6.2±6.1	0.97±0.09
